# The Effect of Complementary and Alternative Medicine on Subfertile Women with *In Vitro* Fertilization

**DOI:** 10.1155/2014/419425

**Published:** 2014-01-16

**Authors:** Yuehui Zhang, Yiman Fu, Fengjuan Han, Hongying Kuang, Min Hu, Xiaoke Wu

**Affiliations:** ^1^Department of Obstetrics and Gynecology, First Affiliated Hospital, Heilongjiang University of Chinese Medicine, Harbin 150040, China; ^2^Center for Post-Doctoral Studies, Heilongjiang University of Chinese Medicine, Harbin 150040, China; ^3^Department of Obstetrics and Gynecology, Heilongjiang University of Chinese Medicine, Harbin 150040, China

## Abstract

About 10–15% of couples have difficulty conceiving at some point in their reproductive lives and thus have to seek specialist fertility care. One of the most commonly used treatment options is *in vitro* fertilization (IVF) and its related expansions. Despite many recent technological advances, the average IVF live birth rate per single initiated cycle is still only 30%. Consequently, there is a need to find new therapies to promote the efficiency of the procedure. Many patients have turned to complementary and alternative medical (CAM) treatments as an adjuvant therapy to improve their chances of success when they undergo IVF treatment. At present, several CAM methods have been used in infertile couples with IVF, which has achieved obvious effects. However, biologically plausible mechanisms of the action of CAM for IVF have not been systematically reviewed. This review briefly summarizes the current progress of the impact of CAM on the outcomes of IVF and introduces the mechanisms.

## 1. Introduction

With rapid economic development, lifestyle changes, and increased environmental pollution, the incidence of infertility has gained increased worldwide attention. Almost 10–15% of couples have suffered from infertility and seek specialist fertility care worldwide [1]. *In vitro* fertilization-embryo transfer (IVF-ET) is the most commonly used treatment option for these couples [2]. For many people, it provides the last possibility for pregnancy. Despite many recent technological advances, the average IVF live birth rate per single initiated cycle is still only 30% [3]. At the same time, the latent safety problems associated with using large doses of ovulation stimulants to obtain more eggs for IVF cannot be ignored [4]. Furthermore, IVF is an expensive procedure, and some couples can afford only a limited number of treatments. Repeated cycles will place enormous economic pressure on the patients. Consequently, there is a need to maximize the efficiency of the procedure [5]. Many patients have turned to complementary and alternative medical (CAM) treatments as an adjuvant therapy to improve their chances of success when they undergo IVF treatment [6–8].

CAM has been defined as diagnosis, treatment, and/or prevention which complements mainstream medicine by contributing to a common whole, satisfying a demand not met by orthodoxy or diversifying the conceptual frameworks of medicine [9]. Recently, complementary and alternative medicine (CAM) modalities have become a popular therapy all over the world as a health care option due to the apparent acceptance of naturalness and harmlessness of CAM [10, 11] and the dissatisfaction with problems associated with conventional medicine [6, 7].

CAM mainly contains the following methods: (i) alternative medical system: TCM (traditional Chinese medicine), Ayurveda, and homeopathy; (ii) mind-body intervention: meditation and biofeedback; (iii) biologically based therapies: herbal therapy and special diet therapy; (iv) manipulative and body-based methods: chiropractic and massage; and (v) energy therapies [12]. At present, several CAM methods have been used in infertile couples with IVF, which has acquired obvious consequences. However, biologically plausible mechanisms of action of CAM for IVF have not been systematically reviewed. This review briefly summarizes the current progress of the impact of CAM on the outcomes of IVF and introduces the mechanisms.

## 2. The Utilization of Acupuncture and Moxibustion in Subfertile Women with IVF-ET 

Acupuncture and moxibustion treatment are one of the traditional Chinese practices widely used in China and some Asian countries. In China, practitioners of acupuncture and moxibusion regard the human body as a whole network based on the theories of meridian, viscera, and Qi-Blood. Acupuncture is a therapy of inserting, manipulating, and retaining very fine needles in specific “acupoints” and has been used in China for centuries to regulate the female reproductive system [13]. The general theory of acupuncture is based on the theory of TCM which believes that there is a kind of energy flow (Qi and Blood) through the body; the balance of this energy flow (Qi and Blood) is essential for health; otherwise disease will follow. Acupuncture was suggested to be capable of correcting imbalances of energy flow (Qi and Blood) by inserting needles to some identified points on the skin. Actually the modern concept of acupuncture has been developed by integrating TCM and modern evidence-based medicine, and the acupuncture therapy is now accepted by many countries worldwide in addition to Asian countries as a kind of therapy of CAM. More and more infertility couples choose acupuncture as an adjunctive treatment to improve IVF outcome [14, 15].

Some studies show that acupuncture is helpful in improving the success rate of assisted reproductive techniques. Some potential mechanisms for its effects on fertility have been proposed [16–18]: (1) acupuncture may mediate the release of neurotransmitters, which may influence the menstrual cycle, ovulation, and fertility by stimulating the secretion of gonadotrophin-releasing hormone; (2) acupuncture may promote blood flow to the uterus by inhibiting uterine central sympathetic nerve activity; (3) acupuncture can alleviate anxiety and stress of infertility patients. All these mechanisms have shown beneficial at the time of embryo transfer to improve the clinical pregnancy rate after IVF. We will discuss these points in the following sections.

### 2.1. The Utilization of Acupuncture in Subfertile Women with IVF-ET

#### 2.1.1. The Impact on the Outcome of Pregnancy in Subfertile Women with IVF-ET Using Acupuncture

In 2002, Paulus et al. first published the results of the randomized controlled trial investigating the effects of acupuncture on pregnancy rates of IVF patients. In this trial, 160 healthy women undergoing IVF or intracytoplasmic sperm injection (ICSI) were randomized to receive acupuncture or no acupuncture. Acupuncture was administered 25 minutes before and after ET. Higher pregnancy rates were found in the acupuncture group compared with the group that did not undergo acupuncture (42.5% versus 26.3%; *P* < 0.03) [19]. Since then numerous randomized controlled trials [20–23] and cohort studies [24–27] proved this finding that acupuncture had a positive impact on reproductive outcomes for IVF patients. Manheimer et al. also demonstrated that adjuvant treatment with acupuncture for IVF patients resulted in a 10% improvement in reproductive outcomes [28]. As a result, more and more infertile patients were treated with acupuncture, and a number of patients who undergo IVF were advised by specialists to receive an adjuvant treatment with acupuncture [29, 30]. Some randomized, controlled, prospective studies demonstrated higher pregnancy rates in the acupuncture group than in the control group [19–22], even though the differences did not reach statistical significance in one study, while the acupuncture subjects who achieved higher pregnancy rates than in the control group (31% versus 23%) in this study [21]. Magarelli and Cridennda carried out a retrospective clinical study and reported higher pregnancy rates (51% versus 36%, *P* < 0.05) and lower miscarriage rates (8% versus 20%, *P* < 0.05) among those subjects who received acupuncture compared with subjects in the control group [24]. Another retrospective study focused on the impact of acupuncture in subjects with poor responders to IVF found out a significant increase in the pregnancy rate in the acupuncture group than in the control group (53% versus 38%, *P* < 0.01) [25]. Dieterle et al. also demonstrated the benefit of acupuncture on clinical pregnancy and ongoing pregnancy rates [22]. Except common acupuncture, electroacupuncture (EA) is another style of acupuncture commonly used in the clinic in China. EA is a therapy where the needle was inserted into the acupoints, inputting currents flow in the near human body bioelectricity. EA combines two stimulations of needle and electric and has some advantages, such as could continuous operation for a long time compared with hand-acupuncture (operate acupuncture with hand), being able to objectively control the amount of stimulation and play an exciting role, and at the same being able to promote blood circulation and improve tissue's nutrition. Cui et al. study confirmed that patients of IVF-ET accepted the intervention of EA, their egg quality can be improved, and the pregnancy rate was increased [31, 32]. Stener-Victorin conducted a randomized controlled multicenter and prospective study; the results confirmed that the EA group achieved a higher implantation rate, pregnancy rate, and birth rate than control group in these patients undergoing IVF-ET [33].

Although some evidence exists on the beneficial effects of acupuncture on IVF success rates, there are still some controversies about the benefits from acupuncture treatment. Some recent clinical trials have shown negative results. Andersen et al. administrated real or placebo acupuncture on the day of ET in 635 patients and found no difference of pregnancy rates in the two groups and implied that the effect of acupuncture in pregnancy rates was only a placebo effect [34]. So et al. conducted a randomized double-blinded comparison between real and placebo acupuncture on IVF outcomes, in this trial, both groups received the acupuncture in pre-transplant of the embryo, and found that the placebo group achieved significantly higher pregnancy rates than real group (55.1% versus 43.8%; *P* < 0.038); furthermore, there were no differences in ongoing pregnancy rates and live birth rates in the two groups [35], and the same results were obtained in these patients undergoing frozen-thawed embryo transfer (FET) cycles [36]. Madaschi et al. also did not find increased pregnancy rates in patients who received acupuncture before and after ET and indicated that the use of acupuncture in patients undergoing IVF was not associated with an increase in pregnancy rates [37]. While, some other studies have proven that, although acupuncture could not change pregnancy rate, acupuncture could improve oocyte and embryo quality [34, 38, 39]. Madaschi et al. found that there was no influence of acupuncture treatment on clinical outcomes between acupuncture group and control group; however, when cycles in which the causes of infertility were exclusively tubal-uterine or idiopathic were evaluated separately, a positive influence of acupuncture on pregnancy (OR = 5.15, 95% CI 1.03 to 34.5; *P* = 0.048) was noted [37].

In addition to the efficacy of acupuncture therapy for IVF-ET patients, the security of acupuncture therapy for IVF-ET patients is also arousing researchers' much attention. The risks of administrating acupuncture during the period of IVF-ET have been widely investigated and have been proved to be minimal [40–43]. Acupuncture has been confirmed to be safe for women undergoing ET [35, 44]. At the same time, some other clinical and experimental evidence suggests that, if the acupuncture is administered by a trained acupuncturist, there will be more safety [45–47]. Stener-Victorin et al. also confirmed this point; they demonstrated that acupuncture can be used to control pain for IVF-ET patients as conventional analgesics without any observed negative side effects [33]. We have listed some clinical trials in Table 1.

#### 2.1.2. The Mechanisms of Acupuncture to Improve the Outcome of Pregnancy of IVF-ET

Regarding the potential mechanisms of acupuncture effect on fertility, the following points deserve careful consideration. The first point is that acupuncture may mediate the release of neurotransmitters, which may influence the menstrual cycle, ovulation, and fertility by stimulating the secretion of gonadotrophin-releasing hormone. Acupuncture was well known for its effect of releasing *β*-endorphin and enkephalin in the central nervous system, and *β*-endorphin levels could influence the secretion of steroid hormones, regulate the menstrual cycle and ovulation [48, 49], and reduce anxiety of infertile couples [50, 51]. Both acupuncture and EA have been proved that could increase the levels of several neurotransmitters such as *β*-endorphin inhibiting the perception of pain and playing a positive effect on the outcomes of IVF [48, 52]. Some authors have shown that acupuncture may influence plasma levels of follicular stimulating hormone, luteinizing hormone, oestradiol, and progesterone, improving ovulation rates [29, 30]. These results have also been proven by animal studies that acupuncture can normalize the secretion of GnRH and influence peripheral gonadotrophin levels [53, 54].

The second point is that acupuncture may promote blood flow to the uterus by inhibiting uterine central sympathetic nerve activity and may promote the endometrial receptivity. A successful implantation of human embryos into the uterine cavity was effected by several important parameters, such as endometrial thickness, morphology, and uterine artery blood flow. High arterial blood flow impedance and low uterine perfusion result in low implantation rate [55–57]. Acupuncture can contribute to the reduction of impedance of the uterine artery by inhibiting central sympathetic nerve and then improve the blood flow to the uterus [48, 50]. EA also can reduce the blood flow impedance index of uterine artery of infertile women and increase the blood perfusion of the endometrium, to provide some good conditions for embryo implantation and improve pregnancy rate. Studies have reported that EA could increase uterine blood flow in infertile women [58, 59]. Stener-Victorin et al. demonstrated positive effects of EA on the pulsatility indices (PIs) of IVF patients and further found higher pregnancy rates (45.9% versus 28.3%; *P* < 0.05) in the EA group compared with a group that used conventional analgesics during oocyte aspiration [59]. In addition, some authors have confirmed that acupuncture also may increase blood flow to the ovaries and reduce ovarian volume and the number of ovarian cysts in polycystic ovarian syndrome (PCOS) [60]. Stener-Victorin et al. reported that low frequency EA was effective in modulating the ovarian blood flow. So we speculated that this may be a potential mechanism of acupuncture leading to positive effects on the outcomes of IVF-ET for subfertile patients with PCOS [61, 62]. Except the blood flow of the uterus, the endometrial receptivity is also very important for the outcomes of IVF-ET, and there is a tight relation between blood flow and endometrial receptivity. Optimal endometrial circulation is the most important factor for endometrial receptivity and favorable reproductive outcome, such as increased blood flow that increases endometrial receptivity [16, 48]. A previous study found that repeated EA treatment may improve endometrial receptivity by decreasing the pulsatility index in the uterine arteries. There is some evidence showing that acupuncture stimulates uterine blood flow, leading to the enhancement of uterine receptivity [59]. Other authors considered that acupuncture may play a key role in implantation by regulating some useful cytokines' expression, such as integrin, leukemia inhibitory factor, stem cell factor, and heparin-binding epidermal growth factor-like growth factor, thereby regulating the time window of implantation [39, 63–65]. An animal study demonstrates that manipulating acupuncture at Zusanli and Sanyinjiao can increase the expression level of vascular endothelial growth factor (VEGF) in endometrium, contributing to successful embryo implantation [66].

Clinicians generally considered that acupuncture in early pregnancy will cause a strong contraction of the uterus, leading to miscarriage. “Acupuncture Figure of Bronze Acupoints (medical records of acupuncture in the ancient China)” also has a medical record that documented that needling the acupoints of Hegu and Sanyinjiao could cause abortion. But, some clinical and animal studies gained exactly the opposite results in recent. They demonstrated a lower miscarriage rate in those patients treated with acupuncture than those patients treated without acupuncture during the period of IVF. Some studies have supported that one possible mechanism of acupuncture on IVF outcomes is to decrease the miscarriage rate in those patients treated with acupuncture than those patients treated without acupuncture during the period of IVF [25–27]. The animal result also confirmed this benefit that acupuncture could inhibit uterine motility in pregnant rats by suppressing the COX-2 enzyme, and thus promoting embryo implantation [67]. This might be the mechanism related to the lower miscarriage rates leading by acupuncture treatment.

The last point is that acupuncture can alleviate anxiety and stress of infertility patients. Psychological stress or stressful life events may negatively influence the clinical pregnancy rates [68, 69]. The wait for the outcome of treatment and uncertainty of success both cause stress and anxiety and can negatively affect the results of IVF [70–72]. Facchinetti et al. demonstrated that the more stress associated, the worse outcome of IVF-ET treatment [73]. Some research suggested that adjuvant acupuncture may help IVF patients handle the psychological and emotional issues when they received subfertility and IVF treatment [74–77]. Smeenk et al. [78] examined urinary levels of stress hormones, adrenaline, noradrenalin, and CORT during treatment and then concluded that the adrenal hormone may be involved in the complex relationship between psychosocial stress and IVF outcomes and further confirmed that there was a greater success for women with lower adrenal hormone compared with women with higher adrenal hormone during undergoing their first IVF cycle. Harlow et al. found that women undergoing IVF treatments were more anxious and it correlated with levels of PRL and cortisol (CORT) [79]; the same results were proved by Ozaki et al. and Merari et al. [80, 81]. Speculating, both PRL and CORT were indicators of stress for IVF-ET patients. The CORT hypersecretion has been reported in women undergoing IVF and ET who fail to achieve implantation. Balk et al. showed that acupuncture was associated with less stress both before and after embryo transfer, and it possibly improved pregnancy rates [82]. Acupuncture may play a role in an improved pregnancy rate by inducing biochemical changes in CORT and PRL during the gonadotropins stimulation in the IVF treatment cycle and decreasing perceived stress at the time of embryo transfer [82]. In addition, the use of acupuncture makes the subject undergoing IVF more relaxed and optimistic, which might be another mechanism related to the high pregnancy rates [38, 82].

In summary, even though numerous studies have been performed in this area, the discrepancy between the positive outcomes and negative outcomes about acupuncture's effect on patients undergoing IVF-ET was still unknown. The possible reasons for these different outcomes might be related to differences in the study design and acupuncture protocol. Another possible reason is related to the number of studied patients being small, and hence the power of the findings is too low for the results to be considered reliable. A study with a larger number of patients was thus needed to clarify this point.

### 2.2. Moxibustion and Its Mechanism of Action in Subfertile Women with IVF-ET

Moxibustion originated from the periods of spring and autumn and the Warring States in ancient China. Moxibustion could warm meridians, relieve pain, and promote blood circulation by burning compressed herbal material at the acupoints. Modern studies confirmed that moxibustion can regulate functional activity of whole body's organs and enhance immune function, increasing the chance of pregnancy [83]. Related studies have confirmed that moxibustion can improve pelvic blood circulation and increase pelvic blood perfusion, regulate the function of hypothalamic-pituitary-gonadal axis and the secretion of steroid hormones, and promote the follicular development and pregnancy rate [84, 85]. A recent prospective, randomized controlled clinical trial indicates that, when embryo implantation has failed, use of acupuncture and moxibustion as adjuvant treatments in women undergoing IVF significantly improves pregnancy outcomes. This study was conducted with 84 infertile patients who had had at least two unsuccessful attempts of IVF. The patients were randomized in control, sham, or acupuncture group. Acupuncture was performed on the first and seventh day of ovulation induction, on the day before ovarian puncture, and on the day after embryo transfer. In the acupuncture group, patients were treated with moxibustion and needling. In the sham group needles were inserted in the areas that did not correspond to known acupuncture points. As a result, the clinical pregnancy rate in the acupuncture group was significantly higher than that in the control and sham groups (35.7% versus 7.1% versus 10.7%; *P* = 0.0169) [86].

## 3. TCM Utilization in Subfertile Women with IVF-ET

Chinese medicine is a medical system that has existed for an estimated 3000 years. Near the turn of the last century, the Chinese systematized various Chinese medicine practices into one unified medical system that could best be integrated with Western medicine and called it traditional Chinese medicine (TCM). TCM includes a series of traditional medical practices originating in China. It is considered as a complementary and alternative medical system in most of the Western countries while remaining as a form of primary care throughout most of Asian countries. TCM is a comprehensive system for the assessment, as well as for the preventative health care and maintenance. TCM theory is extremely complex and originated thousands of years ago through meticulous observation of nature, the cosmos, and the human body. The major theories of TCM include the Yin-yang, the Five Elements, Qi and Blood, and Zang-fu organ theories. In TCM, the understanding of the human body is based on the holistic understanding of the universe as described in Daoism, and the treatment of illness is based primarily on the diagnosis and differentiation of syndromes. The typical TCM therapies include Chinese herbal medicine (CHM) and acupuncture. CHM acts on Zang-fu organs internally, and acupuncture is accomplished by stimulating certain areas of the external body.

Some studies show that 17% of the couples had utilized herbal therapy for infertility in the United States and 46% of patients undergoing IVF admitted regular use of CHM in Irish [42, 43]. Chinese medicine has played a unique advantage to improve egg quality and ovarian response, enable a reduction in the dose of gonadotrophin, increase pregnancy rate, and reduce the incidence of OHSS [87, 88]. We reviewed numbers of the literatures related to treatments for infertility with CHM at the past few years and found out that Chinese medicine therapies mainly included two administration routes, internal administration route and external administration route. Furthermore, internal administration route contained oral administration of Chinese herbal medicine and dietetic therapy of Chinese herbal medicine; external administration route mainly contained enema administration with Chinese herbal medicine. We summarized these as follows.

### 3.1. Oral Administration of CHM in Subfertile Women Undergoing IVF-ET 

#### 3.1.1. The Effects of Combined Treatment with CHM on the Outcomes of IVF-ET

Recently, some RCTs have shown that adjuvant treatment with CHM during IVF-ET could significantly increase pregnancy rate [89–108]. There have a different effect on pregnancy outcome for those patients undergoing IVT-ET when adjuvant treatment with CHM in the different time points of IVF-ET. Some studies have shown that the patients undergoing IVF-ET can get higher pregnancy rate from CHM treatment if they received earlier treatment with CHM [87, 109]. Chen et al. found that treatment with CHM before IVF-ET could significantly increase the implantation rate and live birth rate compared with control group without CHM treatment [110]. Liu et al. randomized 82 cases of endometriosis (EM) who will undergo IVF-ET to CHM group and control group; CHM group received 3-month treatment with CHM before IVF-ET; control group did not receive any treatment before IVF-ET; results showed that CHM could improve the oocyte quality and embryo quality and resulted in a higher pregnancy rate in CHM group than in the control group [98]. The same results were found by Deng et al.; they demonstrated that treatment with CHM before 3 months of IVF-ET could significantly increase the implantation rate and pregnancy rate [97]. In addition, some studies proved that adjuvant treatment of CHM could improve the implantation rates of IVF-ET [97, 111–113]. Luan and Wu applied to an adjuvant treatment with CHM or without CHM in 400 patients undergoing IVF-ET and found that implantation rate, pregnancy rate and live birth rate were significantly higher in CHM group than in the control group [89]. Sun et al. also found the same results in 160 subjects undergoing IVF-ET; there were significantly higher of implantation rate, pregnancy rate, and live birth rate in CHM group than in the control group [93]. Ge et al. randomized 207 cases of IVF-ET to CHM group and control group; CHM group received Cuhuangti Granule (a kind of CHM) for 1 week during the period of downregulating the function of pituitary using GnRH-a, and Jinghou Zengzhi (a kind of CHM) Granule was added to treatment from the day of promoting ovulation using gonadotropin to the day of injecting HCG; then the Cuhuangti Granule was just used for 1 week after retrieving eggs; control group did not give any CHM treatment. The results showed that the implantation rate and twinning rate were higher in CHM group than in the control group (*P* < 0.05), but there were no difference in pregnancy rate and miscarriage rate between the two groups [114]. At the same time, other authors have shown that treatment with CHM after embryo transfer can significantly reduce the miscarriage rate due to the protective action to the fetus from herbal prescriptions [91, 110, 115, 116]. OHSS (ovarian hyperstimulation syndrome) is a common complication during the period of IVF-ET, adjuvant treatment with CHM during IVF-ET, that not only could increase the pregnancy rate but also could decrease the OHSS rate. Lian et al. and Zhang found that those patients adjuvant treatment with CHM could obtain a lower OHSS rate than those patients without CHM treatment during undergoing IVF-ET (5.56% versus 12.0%, *P* < 0.05; 5.56% versus 10.71%, *P* < 0.05) [87, 96]. We have listed some clinical trials in Table 2.

In addition, some researchers put forward artificial cycle sequential therapies of CHM according to the different stages of the menstrual cycle as a treatment method for IVF patients [117]. Artificial cycle sequential therapies with CHM may play a role by regulating the function of hypothalamic-pituitary-ovarian axis. Numerous studies show that using the artificial cycle sequential therapies of CHM for IVF-ET patients can improve patients' sensitivity to medicine of superovulation, increase the oocytes' number and improve egg' quality, and promote the implantation rate and pregnancy rate [4, 5, 118–127]. The therapies of artificial cycle sequential of CHM for IVF-ET patients as an assisted treatment are different in different research, but all of these are based on the basic theory of traditional Chinese medicine. We have listed some studies' results in Table 3.

Although enormous studies have proved that CHM have a postivve effect on pregnancy outcome for those patients undergoing IVF-ET, there still have some opposite opinions about the benefits from CHM [110, 114, 116, 128]; they argued that adjuvant treatment with CHM for IVF-ET patients could not increase the pregnancy rate. The possible reasons for these different outcomes might be related to differences in the study design and CHM composition. Another possible reason is related to the number of studied patients being small, and the method of double blind was not used in the study design, which may result in some bias. So, some prospective, double-blind, placebo controlled RCTs were thus needed to clarify this point in the near future.

#### 3.1.2. The Mechanisms of Oral Administration of CHM to Improve the Outcome of IVF-ET

There are some potential mechanisms of CHM effect on IVF outcomes, including improving the quality of oocytes and embryos and inhibiting the damage to the fetus from harmful antibodies by strengthening maternal immune action [87, 109, 129]. In addition, some CHMs content rich zinc and manganese may promote the development of fetus [130]. Lian and Li randomized 70 cases of endometriosis (EM) that will undergo IVF-ET to CHM group and control group, CHM group received adjuvant treatment with CHM, and control group did not receive any adjuvant treatment. Results showed that the level of GDF-9 mRNA was higher in granulosa cells in CHM group than in the control group, the fertilization rate was higher, and the quality of embryo was improved in CHM group than in the control group, so they implied that CHM could improve the outcomes of IVF-ET by regulating the mRNA expression of GDF-9 in granulose cells [103]. Chang et al. also found the same results [108]. Another study performed by Lian et al. found that CHM could regulate the level of LIF in follicular fluid and speculated that it is involved in regulating the outcome of IVF-ET [101]. Recent researches pointed out that the mechanism of CHM improved the outcomes of IVF-ET which maybe achieved through enhancing endometrial receptivity [89, 104, 105, 110]. Furthermore, a randomized, double-blinded, placebo-controlled clinical trial was conducted. Sixty-six (66) infertile patients who were to undergo IVF-ET were randomly assigned to either a treatment group or a control group; the treatment group received CHM for 3 menstrual cycles before IVF, and the control group received placebo granules. The high-quality oocyte and embryo rates and clinical pregnancy rate were all higher in the treatment group than those in the control group (*P* < 0.05). The DNA methyltransferases (DNMT1) protein expression in the endometrium was much more abundant in the treatment group than that in the control group (*P* < 0.05). So, the authors speculated that the mechanism of CHM improves the outcomes of IVF-ET and maybe achieved through increasing the level of DNMT1 protein expression after treatment, then enhancing endometrial receptivity [104].

### 3.2. Dietetic Therapy of TCM in Subfertile Women Undergoing IVF-ET

Chinese medicine has a theory of “the same homology both of the medicine and food.” With the development of society, the consciousnesses of modern health concept and health care are constantly strengthened, and diet therapy as a prevention and auxiliary treatment method has been widely accepted by the public. Diet therapy as one of the characteristic therapies of Chinese medicine has played an important role in auxiliary treatment for IVF-ET [131–136].

Patients should prepare for the treatment of IVF-ET before 1-2 months of starting treatment, including physical preparation and psychological preparation. Some dietetic therapies of TCM are suitable to these patients during this period, such as some bone soups made of some kinds of CHM, including DangShen, HuangQi, ShanYao, ShiHu, and so on. In addition, dietetic therapy of TCM could help the growth of the follicles to have a sufficient number, improve the quality of eggs, accelerate the growth of endometrium synchronously, and protect ovarian function and embryos [131–136]. So, some dietetic therapies of TCM were chose by those patients when they were undergoing IVF-ET. Some soups were used during the period of IVF-ET; these soups are made of some kinds of CHM, including Polygonatum, Yam, ramie root, Astragalus, Dendrobium, Cistanche, and so on [131–136]. Modern pharmacological study finds that Yam is rich in diosgenin which contains some synthetic materials necessary for various hormones and can promote the synthesis of hormones, and Polygonatum has some functions of antifatigue, antioxidation, and antiaging [134].

### 3.3. Enema Therapy Using CHM and Its Mechanism in Subfertile Women with IVF-ET

Enema therapy using CHM is also known as anorectal drug delivery method which consists of pouring CHM into the rectum where it remains for four to five hours to make the CHM fully absorbed through the intestinal mucosa to treat some specific diseases. It is often used in the treatment of infertility to improve the pregnancy rate. One study observed 131 cases of IVF-ET failed patients treated with uterine lavage or treated with uterine lavage combined with retention enema of CHM; results showed that the clinical pregnancy rate (48.5% versus 29.2%, *P* < 0.05) was higher in combined group than uterine lavage group [137]. Another study also found the same results and implied that the abnormal intrauterine environment may lead to the declination of embryo implantation rate; the treatment of retention enema of CHM may enhance the pregnancy rate by improving endometrial receptivity [138]. The formula of CHM of retention enema could be adjusted according to conditions of the disease and patient, achieving individualized and targeted treatment. The medicine could be absorbed through the rectal mucosa and directly target the lesion, making the local lesion softened and adhesive tissue dissipated. These were the potential mechanisms of retention enema of CHM.

### 3.4. The Mechanisms of Adjuvant Therapy with CHM in IVF Patients

The goals of adjuvant therapy with CHM are premise to ensure the safety of IVF-ET and maximize regulation of the overall health of patients and alleviate the adverse reaction during the period of IVF-ET treatment, then improving the clinical pregnancy rate and live birth rate. The mechanisms of adjuvant therapy with CHM in IVF patients may be as follows: (1) reduced ovarian blood flow resistance and increased ovarian perfusion, thus promoting the follicular development and improving the quality of oocyte [139], (2) improved the endometrial microcirculation and increased the blood flow of endometrium, promoted endometrial thickened, and improved endometrial receptivity and embryo implantation, leading to higher success rate of embryo implant [140], (3) promoted the decidualization of endometrial cells and enhanced the response to exogenous hormone, improving the pregnancy rate [141, 142], (4) improved the levels of transforming growth factor-*β* 1 (TGF-*β* 1) and steroid hormone in follicular fluid, so as to improve the success rate of IVF-ET [143]. In addition, CHM could achieve a collaborative effect with western medicine, reducing the adverse effects from IVF-ET and reducing psychological pressure of the patients.

## 4. Other Complementary and Alternative Therapies

CHM and acupuncture are commonly used to treat subfertile women with IVF as one of the complementary and alternative therapies; psychotherapy and temperature therapy are also commonly used in IVF-ET treatment as a kind of the complementary and alternative therapies.

### 4.1. Psychological Interventions on Patients of IVF

Infertility patients with IVF-ET treatment are special groups; they suffer several pressures from family, society, high cost of medical care, and urgent desire of pregnancy, which easily leads to stress, anxiety, and depression. All of these bad emotions will cause reproductive endocrine dysfunction, leading to adverse effect on the development and maturation of follicles and the outcomes of medical care [144].

We should take some actions to reduce psychological pressure and increase compliance of patients undergoing IVF-ET, in order to improve the success rate of IVF-ET. The psychological interventions were widely used to relieve the pressure of patients as a popular CAM therapy. Psychological interventions include psychological counseling, supportive psychotherapy, the insight therapy or reasoning treatment, beliefs therapy, relaxation therapy, systematic desensitization therapy, behavioral therapy, and group therapy. Psychological interventions are varied, while the relaxation therapy is the usual recommended. Relaxation therapy leads to smooth emotion by relaxing the body step by step. All of these interventions belong to relaxation therapy, such as Chinese qigong, Indian yoga, Japanese meditation, German autogenic training, and American progressive relaxation training. Some studies suggested that psychological intervention can reduce the anxiety and depression of those patients undergoing IVF-ET, relieve the negative impacts on psychology, and improve the pregnancy rate than those patients who did not receive psychological intervention [145–155]. Although there were some evidences that can prove the higher pregnancy rate by psychological intervention, some researchers disagreed with this point; they believed that there is no evidence that could prove the higher pregnancy rate by psychological intervention [156–158]. We have listed some studies in Table 4.

### 4.2. Temperature Therapy on Subfertility Patients with IVF

Temperature is the key factor which influences the outcomes of IVF-ET, but what temperature is suitable for IVF remains controversial. Temperature therapy focuses on finding the most suitable temperature to promote the growth and development of the fertilized egg by adjusting the temperature during the period of IVF-ET. The temperature is different in different stages of IVF-ET, including ovary transfer temperature, oocyte maturation temperature, fertilization and embryo culture temperature, operating temperature of gamete and embryo *in vitro*, and so on. We summarized the effects and its possible mechanisms of action of the temperature therapy on IVF-ET in animals as follows.

(I) Ovarian transport temperature: ovarian *in vitro* should be kept in best temperature which is close to body temperature (36–39°C) or above room temperature, which can reduce oocyte damage from temperature change and has a benefit to the follow-up progress of *in vitro* fertilization [159]. There are some different opinions about that ovarian transport temperature. Sun et al. thought that the transport temperature should be close to room temperature (27 ± 2)°C; it would be more conducive to the maturation and fertilization of oocytes *in vitro* [160]. (II) Egg maturation temperature: it is generally accepted that oocyte maturation temperature is close to the normal body temperature. Liu have shown that 38.5°C is the best oocyte maturation temperature; the higher or lower temperature both will affect the egg maturation rate [161]. (III) Fertilization temperature: some studies have shown that, in a certain temperature range, with the fertilized temperature increasing, the penetration of sperm oocyte and fertilization rate would increase correspondingly [162, 163]. (IV) Embryo culture temperature: one study found that 39°C is the best temperature to the development of fertilized egg *in vitro* [164]. (V) Operating temperature of gametes and embryos *in vitro*: in this process, gametes and embryos are thoroughly exposed to the laboratory environment, which need shorter operation time, larger temperature change, and higher requirements for laboratory technicians. In accordance with the theoretical calculations, the optimum laboratory temperature for operation of gametes and embryos *in vitro* should be 37°C. However, IVF process is not completed in this temperature in the clinic. So far, there are still no reports in the literature about which temperature is more appropriate fertilization temperature; it needs further study.

## 5. Summary

Complementary and alternative medicine has been widely accepted in many Western countries; more and more infertile women select CAM as an adjuvant treatment to promote the pregnancy rate of IVF-ET treatment; Chinese herbal medicine and acupuncture are the most commonly used as the main therapies of TCM. In addition to TCM, there are psychological therapies, temperature therapies, and other alternative therapies which were used for the treatment of IVF-ET. All of these therapies of CAM can contribute to improve the pregnancy rate of IVF-ET patients in different degrees. Although all of current studies about CAM therapies are in the initial stage, there are many shortcomings, such as small size, low quality, and lack of uniform standard in clinical trial; superficial and unsystematic research for the mechanism in the experiment cannot provide a high-quality evidence for clinical application. Therefore, CAM therapy as a promising therapy for the patients with IVF-ET treatment is worthy of further research in the near future.

## Figures and Tables

**Table 1:** Summary of randomized studies of the effect of acupuncture on IVF outcomes.

Study ID	Design	Sample size	Interventions	Outcomes	Limitation
20	RCT	273	Treatment arm: acupuncture intervention Control arm: no intervention	Treatment arm: PR, 39% [37 of 95] Control arm: PR, 26% [21 of 87]	Not mentioned blindness Small sample size

19	RCT	160	Treatment arm: acupuncture intervention Control arm: no intervention	Treatment arm: PR, 42.5% [34 of 80] Control arm: PR, 26.3% [21 of 80]^*※*^	Not mentioned blindness Small sample size

21	Single-blind, RCT	228	Treatment arm: acupuncture intervention Control arm: no intervention	Treatment arm: PR, 31% [33 of 107] Control arm: PR, 23% [26 of 114]	Single-blind trial Small sample size

22	RCT	225	Treatment arm: acupuncture intervention Control arm: no intervention	Treatment arm: PR, 33.6% [39 of 116] Control arm: PR, 15.6% [17 of 109]^*※*^	Not mentioned blindness Small sample size

37	Single-blind, RCT	150	Treatment arm: acupuncture intervention Control arm: no intervention	Treatment arm: PR, 50% [39 of 78] Control arm: PR, 42.6% [29 of 68]	Single-blind trial Small sample size

59	RCT	44	Treatment arm: acupuncture intervention Control arm: no intervention	Treatment arm: PR, 30% [9 of 30] Control arm: PR, 28.6% [4 of 14]	Not mentioned blindness Small sample size

35	Double-blind, RCT	370	Treatment arm: acupuncture intervention Control arm: placebo acupuncture intervention	Treatment arm: PR, 55.1 [102 of 185]^#^ Control arm: PR, 43.8% [81 of 185]	

36	Double-blind, RCT	226	Treatment arm: acupuncture intervention Control arm: placebo acupuncture intervention	There were no significant differences in outcomes of PR, OPR, LBR, and IR in the placebo acupuncture group than in the real acupuncture group	Small sample size

34	Double-blinded, RCT	635	Treatment arm: acupuncture intervention Control arm: placebo acupuncture intervention	There were no significant differences in outcomes of OPR and LBR and IR between the placebo acupuncture group and the real acupuncture group	

39	RCT	416	Treatment arm: acupuncture intervention Control arm: no intervention	There was no significantly increased PR in the acupuncture group than in the control group	Not mentioned blindness

44	Double-blinded, RCT	160	Treatment arm: acupuncture intervention Control arm: placebo acupuncture intervention	CPR had no significant difference between the true acupuncture group and the sham acupuncture group	Small sample size

32	RCT	66	Treatment arm: acupuncture intervention Control arm: no intervention	The fertilization rate, cleavage rate, and the rate of high-quality embryos were all significantly higher in the acupuncture group than in the control group	Not mentioned blindness Small sample size

Note: RCT: randomized clinical trial; PR: pregnancy rate; OPR: ongoing pregnancy rate; LBR: live birth rate; IR: implantation rate; FR: fertilization rate; CR: cleavage rate.^*※*^ *P* < 0.05 versus treatment arm; ^#^ *P* < 0.05 versus control arm.

**Table 2:** Summary of the effect of randomized studies of CHM on IVF outcomes.

Study ID	Design	Sample size	Interventions	Outcomes	Composition	Limitation
90	RCT	400	Treatment arm: CHM Control arm: no intervention	Treatment arm: PR, 59.41% [101 of 170] Control arm: PR, 46.79% [80 of 171]^*※*^	(1) Tiaojing Zhuyun pill: Cornu cervi (Lurong), Epimedium (Yinyanghuo), Curculigo orchioides (Xianmao), Radix dipsaci (Xuduan), Herba taxilli (Shangjisheng), Dodder (Tusizi), Fructus Lycii (Gouqizi), Fructus rubi (Fupenzi),Yam (Shanyao), Lotus seed (Lianzi), Poria cocos (Fuling), Astragalus (Huangqi), White paeony root (Baishao), Semen ziziphi pinosae (Suanzaoren), Uncaria (Gouteng), Salviae miltiorrhizae (Danshen), Red peony root (Chishao), and Caulis Spatholobi (Jixueteng) (2) Liuwei Dihuang pill: Prepared rehmannia root (Shudihuang), Pulp of cornus (Shanyurou), Yam (Shanyao), Rhizoma alismatis (Zexie), Cortex moutan (Danpi), and Poria cocos (Fuling) (3) Xiaoyao pill: Bupleurum (Chuaihu), Angelica sinensis (Danggui), White paeony root (Baishao), Atractylodes (Baizhu), Poria cocos (Fuling), Mechanism (Bohe), Ginger (Shengjiang), and Liquorice (Gancao) (4) Gongyan Kang Granule: Angelica sinensis (Danggui), Red peony root (Chishao), North defeated sauce (Beibaijiang), Rhizoma cyperi (Xiangfu), Roasted ginger (Paojiang), Lycopus lucidus (Zelan), Ligusticum wallichii (Chuanxiong), Safflower (Honghua), Bupleurum (Chuaihu), Semen plantaginis (Cheqianzi), Alga (Haizao), and Rhizoma corydalis (Yanhusuo)	Not mentioned blindness

91	RCT	244	Treatment arm: CHM Control arm: no intervention	Treatment arm: PR, 47.37% [36 of 76] Control arm: PR, 32.14% [54 of 168]^*※*^	(1) Zhongyu I: Herba leonuri (Yimucao), Prepared rehmannia root (Shudihuang), Angelica sinensis (Danggui), Ligusticum wallichii (Chuanxiong), Dodder (Tusizi), Fructus Lycii (Gouqizi), Fructus rubi (Fupenzi), Rhizoma cyperi (Xiangfu), Salviae miltiorrhizae (Danshen), Atractylodes (Baizhu), and White paeony root (Baishao) (2) Zhongyu II: Cinnamon (Rougui), Radix codonopsis (Dangshen), Astragalus (Huangqi), Yam (shanyao), Epimedium (Yinyanghuo), Dodder (Tusizi), Fructus Lycii (Gouqizi), Cistanche salsa (Roucongrong), Morindaofficinalis (Bajitian), Prepared rehmannia root (Shudihuang), and Angelica sinensis (Danggui) (3) Gutai decoction: Prepared rehmannia root (Shudihuang), Yam (Shanyao), Pulp of cornus (Shanyurou), Dodder (Tusizi), Fructus Lycii (gouqizi), Radix codonopsis (Dangshen), Radix dipsaci (Xuduan), Herba taxilli (Sangjisheng), Atractylodes (Baizhu), White paeony root (Baishao), and Liquorice (Gancao)	Not mentioned blindness and drop-out rate

93	RCT	220	Treatment arm: CHM Control arm: no intervention	Treatment arm: PR, 52.73% [58 of 110] Control arm: PR, 29.09% [32 of 110]^*※*^	(1) Yishen Angong I: Dried radix rehmanniae (Shengdihuang), Prepared rehmannia root (Shudihuang), White paeony root (Baishao), Yam (Shanyao), Radix dipsaci (Xuduan), Radix scutellariae (Huangqin), Herba taxilli (Shangjisheng), Gelatin beads (Ejiaozhu), Esliptae herba (Hanliancao), and Liquorice (Gancao) (2) Yishen Angong II: Radix codonopsis (Dangshen), Atractylodes (Baizhu), Radix dipsaci (Xuduan), Herba taxilli (Shangjisheng), Eucommia (Duzhong), Dodder (Tusizi), White paeony root (Baishao), Prepared rehmannia root (Shudihuang), Poria cocos (Fuling), Gelatin beads (Ejiaozhu), and Liquorice (Gancao)	Not mentioned blindness and drop-out rate

94	RCT	207	Treatment arm: CHM Control arm: no intervention	Treatment arm: PR, 54.46% [55 of 101] Control arm: PR, 50.94% [54 of 106]	(1) Cuhuangti Granule: Prepared rehmannia root (Shudihuang), Yam (shanyao), Fructus Lycii (Gouqizi), Pulp of cornus (Shanyurou), Dodder (Tusizi), Antler gum (Lujiaojiao), Tortoiseshell (Guiban), Radix codonopsis (Dangshen), Atractylodes (Baizhu), Lentil (Biandou), Coix seed (Yiyiren), and Poria cocos (Fuling) (2) Jinghou Zengzhi Granule: Liquorice (Gancao), Radix codonopsis (Dangshen), Angelica sinensis (Danggui), Ligusticum wallichii (Chuanxiong), White paeony root (Baishao), Atractylodes (Baizhu), Poria cocos (Fuling), Dodder (Tusizi), Prepared rehmannia root (Shudihuang), Eucommia (Duzhong), Cornu cervi degelatinatum (Lujiaoshuang), Pulp of cornus (Shanyurou), and Capsicum Annuum (Chuanjiao)	Not mentioned blindness and drop-out rate

95	RCT	200	Treatment arm: CHM Control arm: no intervention	Treatment arm: PR, 45% [45 of 100] Control arm: PR, 39% [39 of 100]	Antai I: Codonopsis pilosula (Dangshen), Dodder (Tusizi), Dogwood (Shanzhuyu), Arable land (Shoudi), Atractylodes (Baizhu), Yam (Shanyao), Dipsacus (Xuduan), Eucommia (Duzhong), Wolfberry (Gouqi), root of herbaceous peony (Baishao), Mistletoe (Sangjisheng), Angelica (Danggui), and Licorice (Gancao)	Not mentioned blindness and drop-out rate

97	RCT	160	Treatment arm: CHM Control arm: no intervention	Treatment arm: PR, 60.0% [48 of 80] Control arm: PR, 38.8% [31 of 80]^*※*^	Bubao Decoction: Dodder (Tusizi), Fructus Lycii (Gouqizi), Polygonatum (Huangjing), Caulis Spatholobi (Jixueteng), Fallopia multiflora (Heshouwu), Prepared rehmannia root (Shudihuang), Tortoise-plastronglue (Guibanjiao), Antler gum (Lujiaojiao), *Morinda officinalis* (Bajitian), Radix dipsaci (Xuduan), Angelica sinensis (Danggui), Epimedium (Yinyanghuo), Human placenta powder (Zihechefen), Liquidambar formosana hance (Lulutong), and Rhizoma cyperi (Xiangfu)	Not mentioned blindness and drop-out rate

98	Single-blind RCT	122	Treatment arm: CHM Control arm: no intervention	Treatment arm: PR, 39.34% [24 of 61] Control arm: PR, 29.51% [18 of 61]	Erzhi Tiangui Granule: Ligustrum (Nvzhenzi), Eclipta (Hanliancao), Medlar (Gouqizi), Dodder (Tusizi), Angelica (Danggui), root of herbaceous peony (Baishao), Dried rehmannia (Shengdihuang), Ligusticum (Chuanxiong), System Cyperus (Xiangfu), and Licorice (Gancao)	Single-blind trial Not mentioned drop-out rate

99	RCT	100	Treatment arm: CHM Control arm: no intervention	Treatment arm: PR, 16.0% [8 of 50] Control arm: PR, 2.0% [1 of 50]^*※*^	Zishen Huoxue decoction: prepared rehmannia root (Shudihuang), Polygonatum (Huangjing), Fructus Lycii (Gouqizi), Pulp of cornus (Shanyurou), Angelica sinensis (Danggui), White paeony root (Baishao), Ligusticum wallichii (Chuanxiong), Salviae miltiorrhizae (Danshen), and Semen persicae (Taoren**)**	Not mentioned blindness and drop-out rate Small sample size

100	RCT	98	Treatment arm: CHM Control arm: no intervention	Treatment arm: PR, 40.74% [21 of 50] Control arm: PR, 26.00% [12 of 48]^*※*^	Bushen Huatan decoction: Amethyst (Zishiying), Eucommia (Duzhong), Dodder (Tusizi), Pinellia ternate (Banxia), Citrus (Chenpi), Atractylodes (Baizhu), Atractylodes (Cangzhu), Rhizoma cyperi (Xiangfu), Aflatoxin (Shenqu), and Ligusticum wallichii (Chuanxiong)	Not mentioned blindness and drop-out rate Small sample size

101	RCT	82	Treatment arm: CHM Control arm: no intervention	Treatment arm: PR, 63.4% [26 of 41] Control arm: PR, 41.5% [17 of 41]^*※*^	(1) Jinghou Zengzhi Granule: Radix codonopsis (dangshen), Atractylodes (baizhu), Poria cocos (fuling), Angelica sinensis (danggui), Prepared rehmannia root (shudihuang), Eucommia (duzhong), and Pulp of cornus (shanyurou) (2) Cuhuangti Granule: Radix codonopsis (Dangshen), Atractylodes (Baizhu), Coix seed (Yiyiren), Prepared rehmannia root (Shudihuang), Yam (Shanyao), and Dodder (Tusizi) (3) Jingqian Granule: Bupleurum (Chai hu), Atractylodes (Baizhu), Angelica sinensis (Danggui), White paeony root Baishao), Safflower (Honghua), and Corydalis (Yuanhu)	Not mentioned blindness and drop-out rate Small sample size

102	RCT	82	Treatment arm: CHM Control arm: no intervention	Treatment arm: PR, 39.34% [17 of 42] Control arm: PR, 20.00% [8 of 40]^*※*^	Quyu Jiedu Granule: Sargentodoxa cuneata (Hongteng), Rose (Meiguihua), Honeysuckle (Jinyinhua), Forsythia (Lianqiao), Salvia (Danshen), Red peony (Chishao), and Moutan (Danpi)	Not mentioned blindness and drop-out rate Small sample size

103	RCT	80	Treatment arm: CHM Control arm: no intervention	Treatment arm: PR, 62% [31 of 50] Control arm: PR, 33% [10 of 30]^*※*^	Shoutai pill: Dodder (Tusizi), Mistletoe (Sangjisheng), Dipsacus (Xuduan), Eucommia (Duzhong), White paeony root (Baishao), Codonopsis pilosula (Dangshen), Astragalus (Huangqi), Atractylodes (Baizhu), Burnet charcoal (Diyutan), Dried rehmannia (Shengdihuang), Eclipta (Hanliancao), Scutellaria (Huangqin), and Licorice (Gancao)	Not mentioned blindness and drop-out rate Small sample size

104	RCT	80	Treatment arm: CHM Control arm: no intervention	Treatment arm: PR, 57.5% [23 of 40] Control arm: PR, 30.0% [12 of 40]^*※*^	Shoutai pill: Dodder (Tusizi), Mistletoe (Sangjisheng), Dipsacus (Xuduan), Hide gelatin (Ejiao), Codonopsis pilosula (Dangshen), Atractylodes (Baizhu), Astragalus (Huangqi), Scutellaria (Huangqin), Antler cream (Lujiaoshuang), Rehmannia (Shoudi), and Amomum (Sharen)	Not mentioned blindness and drop-out rate Small sample size

105	RCT	80	Treatment arm: CHM Control arm: no intervention	Treatment arm: PR, 47.6% [20 of 42] Control arm: PR, 26.3% [15 of 38]^*※*^	Erzhi Tiangui Granule: Medlar (Gouqizi), Dodder (Tusizi), Ligustrum (Nvzhenzi), Eclipta (Hanliancao), Rehmannia (Shoudi), Cyperus (Xiangfu), Angelica (Danggui), White paeony root (Baishao), Ligusticum (Chuanxiong), and Licorice (Gancao)	Not mentioned blindness and drop-out rate Small sample size

106	RCT	72	Treatment arm: CHM Control arm: no intervention	Treatment arm: PR, 83.33% [30 of 36] Control arm: PR, 63.89% [23 of 36]^*※*^	Radix codonopsis (Dangshen), Astragalus (Huangqi), Herba taxilli (Shangjisheng), White paeony root (Baishao**)**, Atractylodes (Baizhu), Dodder (Tusizi), Radix scutellariae (Huangqin). Yam (Shanyao), and Radix dipsaci (Xuduan)	Not mentioned blindness and drop-out rate Small sample size

107	Single-blind RCT	70	Treatment arm: CHM Control arm: no intervention	Treatment arm: PR, 65.7% [23 of 35] Control arm: PR, 49.1% [17 of 35]^*※*^	Dane Fukang Jiangao: Purple Salvia (Zidanshen), Curcuma (Ezhu), Bamboo (Zuye), Bupleurum (Chaihu), Notoginseng (Sanqi), Red peony (Chishao), Angelica (Danggui), Trigonous (Sanleng), Dodder (Tusizi), Cyperus (Xiangfu), Corydalis (Yanhusuo), and Licorice (Gancao)	Single-blind trial Small sample size Not mentioned drop-out rate

88	RCT	64	Treatment arm: CHM Control arm: no intervention	Treatment arm: PR, 36.11% [13 of 36] Control arm: PR, 21.43% [6 of 28]^*※*^	Erzhi Tiangui Granule: Ligustrum lucidum ait (Nvzhenzi), Esliptae herba (Hanliancao), Fructus Lycii (Gouqizi), Dodder (Tusizi), Angelica sinensis (Danggui), White paeony root (Baishao), Ligusticum wallichii (Chuanxiong), Prepared rehmannia root (Shudihuang), Rhizoma cyperi (Xiangfu), and Liquorice (Gancao)	Not mentioned blindness and drop-out rate Small sample size

109	RCT	63	Treatment arm: CHM Control arm: no intervention	Treatment arm: PR, 43.5% [14 of 31] Control arm: PR, 28.8% [9 of 32]^*※*^	(1) CHM decoction I: Ligustrum (Nvzhenzi), Eclipta (Mohanlian), Medlar (Gouqizi), Dried rehmannia (Shengdihuang), Rehmannia (Shoudihuang), Angelica (Danggui), and Root of herbaceous peony (Baishao) (2) CHM decoction II: Rehmannia (Shoudihuang), Dodder (Tusizi), Dipsacus (Xuduan), Mistletoe (Sangjisheng), Eucommia (Duzhong), Morinda (Bajitian), Atractylodes (Baizhu), Angelica (Danggui), Salvia (Danshen), and Scutellaria (Huangqin)	Not mentioned blindness and drop-out rate Small sample size

110	RCT	61	Treatment arm: CHM Control arm: no intervention	Treatment arm: PR, 56.7% [17 of 31] Control arm: PR, 45.2% [14 of 30]^*※*^	Erzhi Daotan decoction: Ligustrum lucidum ait (Nvzhenzi), Esliptae herba (Hanliancao), Epimedium (Yinyanghuo), Angelica sinensis (Danggui), Safflower (Honghua), Atractylodes (Cangzhu), Pinellia ternate (Banxia), Citrus (Chenpi), and Liquorice (Gancao)	Not mentioned blindness and drop-out rate Small sample size

111	RCT	60	Treatment arm: CHM Control arm: no intervention	Treatment arm: PR, 73.33% [22 of 30] Control arm: PR, 46.67% [14 of 30]^*※*^	Bushen Tiaojing decoction: Rehmannia (Shoudihuang), Angelica (Danggui), Yam (Shanyao), Cornus (Shanyurou), Medlar (Gouqizi), Ligustrum (Nvzhenzi), Epimedium (Yinyanghuo), Placenta (Ziheche), Raspberry (Fupenzi), Dodder (Tusizi) Cyperus (Xiangfu), Safflower (Honghua), and root of herbaceous peony (Baishao)	Not mentioned blindness and drop-out rate Small sample size

112	RCT	58	Treatment arm: CHM Control arm: no intervention	Treatment arm: PR, 73.33% [22 of 30] Control arm: PR, 46.42% [13 of 28]^*※*^	Bushen Tiaojing decoction: Rehmannia (Shoudihuang), Angelica (Danggui), Yam (Shanyao), Cornus (Shanyurou), Medlar (Gouqizi), Ligustrum (Nvzhenzi), Epimedium (Yinyanghuo), Placenta (Ziheche), Raspberry (Fupenzi), Dodder (Tusizi), Cyperus (Xiangfu), Safflower (Honghua), root of herbaceous peony (Baishao), Cistanche (Roucongrong), and Salvia (Danshen)	Not mentioned blindness and drop-out rate Small sample size

Note: CHM: Chinese herbal medicine; PR: pregnancy rate; ^*※*^ *P* < 0.05 versus treatment arm.

**Table 3:** Summary of the effect of the clinical studies of artificial cycle sequential therapies with CHM on IVF outcomes.

Study ID	Design	Sample size	Interventions	Outcomes	Composition	Limitation
121	Single-blind RCT	60	Treatment arm: CHM + COH Control arm: COH	Treatment arm: PR, 46.7% [14 of 30] Control arm: PR, 35.3% [10 of 30]	Period: Taohong Siwu decoction Postmenstrual period: Xiaoyao Granule Intermenstrual period: Zuogui pill plus Chinese honeylocust spine (Zao jiao ci) Premenstrual period: Wuzi Yanzong decoction combined with Erxian decoction plus Fluorite (Zi shi ying), fruit of beautiful sweetgum (Lu lu tong), deglued antler powder (Lu jiao shuang), and Indianmulberry (Ba ji tian)	Not mentioned drop-out rate Small sample size

122	RCT	160	Treatment arm: CHM + COH Control arm: COH	Treatment arm: PR, 63.5% [40 of 63] Control arm: PR, 41.5% [27 of 65]^*※*^	Follicular phase: Yueju pill combined with Wuwei Tiaojing Granule Ovulation phase: Bushen Cupailuan decoction Luteal phase: Yulin Zhu combiner with Xiaoyao Granule Day 1-day 14 of ET: Yulin Zhu combiner with Xiaoyao Granule Confirmed pregnancy: Yulin Zhu combiner with Xiaoyao Granule	Not mentioned blindness Small sample size

123	RCT	100	Treatment arm: CHM + COH Control arm: COH	Treatment arm: PR, 50% [25 of 50] Control arm: PR, 32% [16 of 50]^*※*^	The basic prescription: Prepared rehmannia root (Shu di), Chinese angelica root (Dang gui), Buckhorn (Lu jiao), Tortoise plastron (Gui jia), Achyranthes root (Huai niu xi), Motherwort fruit (Chong wei zi), Ganoderma lucidum (Ling zhi), Fruit of barbary wolf berry (Gou qi zi), Dodder (Tu si zi), Bighead atractylodes rhizome (Huai shan yao), Epimeddium (Xian ling pi), Pseudostellaria root (Tai zi shen), Red sage root (Dan shen), Anemarrhena (Zhi mu), Bark of amur corktree (Huang bai), and Dried human placemta (Zi he che) Follicular phase: Prepared rehmannia root (Shu di), Chinese angelica root (Dang gui), Fruit of barbary wolf berry (Gou qi zi), Epimeddium (Xian ling pi), Indianmulberry (Ba ji tian), Buckhorn (Lu jiao), Love ipea seed (Tao ren), Safflower (Hong hua), and Cyperirhizome (Xiang fu) Luteal phase: Dodder (Tu si zi), Indianmulberry (Ba ji tian), and Buckhorn (Lu jiao)	Not mentioned blindness and drop-out rate Small sample size

125	RCT	58	Treatment arm: CHM + COH Control arm: COH	Treatment arm: PR, 26.7% [8 of 30] Control arm: PR, 21.4% [6 of 28]^*※*^	Follicular phase: Motherowrt fruit (Chong wei zi), Red peony root (Chi shao), Fruit of beautiful sweetgum (Lu lu tong), Love ipea seed (Tao ren), Safflower (Hong hua), Achyranthes root (Niu xi), Chinese angelica root (Dang gui), Red sage root (Dan shen), Cyperirhizome (Xiang fu), Lindera aggregate (Wu yao), Alisma rhizome (Ze xie), and Seed of cow-fat (Wang bu liu xing) Ovulation phase: Fruit of glossy privet (Nv zhen zi), Chinese angelica root (Sang gui), Yeradetajo (Han lian cao), Red sage root (Dan shen), Cyperirhizome (Xiang fu), Aucklandia root (Mu xiang), Lindera aggregate (Wu yao), and Alisma rhizome (Ze xie) Luteal phase: Cassia twig (Gui zhi), Epimeddium (Xian ling pi), Glue of tortoise plastrom (Gui ban jiao), Love ipea seed (Tao ren), Safflower (Hong hua), Chinese angelica root (Dang gui), Ed sage root (Dan shen), Cyperirhizome (Xiang fu), Aucklandia root (Mu xiang), Lindera aggregate (Wu yao), and Alisma rhizome (Ze xie)	Not mentioned blindness and drop-out rate Small sample size

126	RCT	42	Treatment arm: CHM + COH Control arm: COH	Treatment arm: PR, 38.1% [8 of 21] Control arm: PR, 19.1% [4 of 21]^*※*^	Follicular phase: Pilose asiabell root (Dang shen), Bighead atractylodes rhizome (Shan yao), Motherowrt fruit (Chong wei zi), Dodder (Tu si zi), Salvia (Dan shen), Cyperirhizome (Xiang fu), Fleece-flower root (He shou wu), Fruit of glossy privet (Nv zhen zi),Prepared rehmannia root (Shu di huang), and Broomrape (Rou cong rong) Ovulatory phase: Ed sage root (Dan shen), Red peony root (Chi shao), Dogwood (Shan zhu yu), Lycopus herb (Ze lan), Cyperirhizome (Xiang fu), Fruit of glossy privet (Nv zhen zi), Dodder (Tu si zi), Aucklandia root (Mu xiang), and Lindera aggregate (Wu yao) Luteal phase: Glue of tortoise plastrom (Gui ban jiao), Dodder (Tu si zi), Loranthus (Sang ji sheng), Broomrape (Rou cong rong), Hinalayan teasel (Xu duan), Epimeddium (xian ling pi), Cyperirhizome (Xiang fu), Curculigo rhizome (Xian mao), and Indianmulberry (Ba ji tian)	Not mentioned blindness and drop-out rate Small sample size

127	RCT	53	Treatment arm: CHM + COH Control arm: COH	Treatment arm: PR, 48.0% [12 of 25] Control arm: PR, 32.1% [9 of 28]^*※*^	The basic prescription: Dodder (Tu si zi), Loranthus (Sang ji sheng), Red peony root (Chi shao), Chinese angelica root (Dang gui), Ed sage root (Dan shen), Cyperirhizome (Xiang fu), Notoginseng (San qi), and Gallus gallus (Ji nei jin) Follicular phase: the basic prescription add Fruit of barbary wolf berry (Gou qi zi), Lilyturf root (Mai dong), Zedoary (E zhu), and Leonurus heterophyllus (Yi mu cao) Ovulatory phase: the basic prescription add herb of diverse wormwood (Liu ji nu), Fruit of beautiful sweetgum (Lu lu tong), and Astragalus root (Huang qi) Luteal phase: the basic prescription add Indianmulberry (Ba ji tian), Fruit of glossy privet (Nv zhen zi), and Prepared rehmannia root (Shu di huang)	Not mentioned blindness and drop-out rate Small sample size

128	Observational studies	480	Treatment arm: CHM	PR, 10.8% [52 of 480]	Period: Cyperirhizome (Xiang fu), Root-bark of peony (Mu dan pi), Fruit of hawthorn (Shan zha), Red sage root (Dan shen), Red peony root (Chi shao), Atractylodes rhizome (Zhi cang zhu), Faces of flying squirrel (Wu ling zhi), Leonurus heterophyllus (Yi mu cao), Indian bread (Fu ling), and Dipsacus root (Chuan duan) Follicular phase: Chinese angelica root (Dang gui), Red peony root (Chi shao), Root of herbaceous peony (Bai shao), Cyperirhizome (Zhi xiang fu), Bighead atractylodes rhizome (Shan yao), Dogwood (Shan zhu yu), Oyster (Mu li), Indian bread (Fu ling), Alisma rhizome (Ze xie), Dipsacus root (Chuan duan), Loranthus (Sang ji sheng), and Fruit of hawthorn (Shan zha) Ovulatory phase: Chinese angelica root (Dang gui), Red peony root (Chi shao), Root of herbaceous peony (Bai shao, Bighead atractylodes rhizome (Shan yao), Dogwood (Shan zhu yu), Root-bark of peony (Dan pi), Indian bread (Fu ling), Dipsacus root (Chuan duan), Buckhorn (Lu jiao), Faces of flying squirrel (Wu ling zhi), Safflower (Hong hua), and Rhizome of Sichuan lovage (Chuan xiong) Luteal phase: Chinese angelica root (dang gui), Root of herbaceous peony (Bai shao), Bighead atractylodes rhizome (Shan yao), Root-bark of peony (Dan pi), Indian bread (Fu ling), Dipsacus root (Chuan duan), Buckhorn (Lu jiao), Fluorite (Zi shi ying), Taurus (Jin ling zi), Corydalis tuber (Yan hu suo), and Fruit of hawthorn (Shan zha)	No control group Not mentioned drop-out rate

Note: CHM: Chinese herbal medicine; COH: control ovarian hyperstimulation; PR: pregnancy rate; ^*※*^ *P* < 0.05 versus treatment arm.

**Table 4:** Summary of the effect of the clinical studies of psychological intervention on IVF outcomes.

Study ID	Design	Sample size	Interventions	Outcomes	Limitation
148	RCT	420	Treatment arm: psychological intervention Control arm: no intervention	Treatment arm: PR, 44.83% [39 of 125] Control arm: PR, 28.95% [22 of 132]^*※*^	

158	Nonrandomized clinical trial	110	Treatment arm: psychological intervention Control arm: no intervention	Treatment arm: PR, 38.18% [21 of 55] Control arm: PR, 23.64% [13 of 55]	Not mentioned drop-out rate Small sample size Not randomized trial

149	RCT	210	Treatment arm: psychological intervention through the treatment Control arm: psychological intervention just in retrieval day, ET day, and pregnancy test day	Treatment arm: PR, 53.33% [56 of 105] Control arm: PR, 39.05% [41 of 105]^*※*^	Not mentioned drop-out rate

150	RCT	286	Treatment arm: psychological intervention Control arm: no intervention	Treatment arm: PR, 40.79% [62 of 152] Control arm: PR, 30.60% [41 of 134]^*※*^	Not mentioned drop-out rate

159	RCT	100	Treatment arm: psychological intervention Control arm: no intervention	Treatment arm: PR, 35.7% [15 of 42] Control arm: PR, 29.8% [14 of 47]	Small sample size

151	RCT	268	Treatment arm: psychological intervention Control arm: no intervention	Treatment arm: PR, 41.67% [80 of 192] Control arm: PR, 28.94% [22 of 76]^*※*^	Not mentioned drop-out rate

152	RCT	207	Treatment arm: psychological intervention Control arm: no intervention	Treatment arm: PR, 44.5% [49 of 108] Control arm: PR, 31% [31 of 99]	Not mentioned drop-out rate

153	RCT	385	Treatment arm: psychological intervention Control arm: no intervention	Treatment arm: PR, 64.56% [122 of 189] Control arm: PR, 53.65% [103 of 192]^*※*^	

154	RCT	218	Treatment arm: IKAP intervention Control arm: healthy education	Treatment arm: PR, 54.0% [65 of 120] Control arm: PR, 39.8% [39 of 98]^*※*^	Not mentioned drop-out rate

155	RCT	447	Treatment arm: psychological intervention Control arm: no intervention	Treatment arm: PR, 43.5% [121 of 278] Control arm: PR, 30.9% [52 of 169]^*※*^	Not mentioned drop-out rate

156	RCT	268	Treatment arm: psychological intervention Control arm: no intervention	Treatment arm: PR, 46.32% [89 of 129] Control arm: PR, 32.89% [25 of 76]^*※*^	

157	RCT	1060	Treatment arm: psychological intervention Control arm: no intervention	Treatment arm: PR, 51.71% [320 of 590] Control arm: PR, 48.88% [240 of 470]^*※*^	Not mentioned drop-out rate

Note: PR: pregnancy rate; IKAP: information-knowledge-attitude-practice; ^*※*^ *P* < 0.05 versus treatment arm.
